# My Action, My Self: Recognition of Self-Created but Visually Unfamiliar Dance-Like Actions From Point-Light Displays

**DOI:** 10.3389/fpsyg.2018.01909

**Published:** 2018-10-16

**Authors:** Bettina E. Bläsing, Odile Sauzet

**Affiliations:** ^1^Neurocognition and Action – Biomechanics Research Group, Faculty of Psychology and Sport Science, Bielefeld University, Bielefeld, Germany; ^2^Center of Excellence Cognitive Interaction Technology (CITEC), Bielefeld University, Bielefeld, Germany; ^3^Bielefeld School of Public Health/AG 3 Epidemiology & International Public Health, Bielefeld University, Bielefeld, Germany; ^4^StatBeCe, Center for Statistics, Bielefeld University, Bielefeld, Germany

**Keywords:** action recognition, self-recognition, motor learning, point-light walker, dance-like actions

## Abstract

Previous research has shown that motor experience of an action can facilitate the visual recognition of that action, even in the absence of visual experience. We conducted an experiment in which participants were presented point-light displays of dance-like actions that had been recorded with the same group of participants during a previous session. The stimuli had been produced with the participant in such a way that each participant experienced a subset of phrases only as observer, learnt two phrases from observation, and created one phrase while blindfolded. The clips presented in the recognition task showed movements that were either unfamiliar, only visually familiar, familiar from observational learning and execution, or self-created while blind-folded (and hence not visually familiar). Participants assigned all types of movements correctly to the respective categories, showing that all three ways of experiencing the movement (observed, learnt through observation and practice, and created blindfolded) resulted in an encoding that was adequate for recognition. Observed movements showed the lowest level of recognition accuracy, whereas the accuracy of assigning blindfolded self-created movements was on the same level as for unfamiliar and learnt movements. Self-recognition was modulated by action recognition, as participants were more likely to identify themselves as the actor in clips they had assigned to the category “created” than in clips they had assigned to the category “learnt,” supporting the idea of an influence of agency on self-recognition.

## Introduction

Human body motion has been studied by many authors using point-light displays in which only white dots on a black background indicate relevant parts (joints) of a moving body (Johansson, 1973). Such displays are used as stimuli that contain only movement information without any additional information about the person (see Thornton, 2006; Blake and Shiffrar, 2007). Studies using point-light walkers have shown that the moving dots representing a body in motion reliably convey information about the person’s familiarity (Cutting and Kozlowski, 1977; Troje et al., 2005), gender (Kozlowski and Cutting, 1977; Pollick et al., 2005), and emotional state (Dittrich et al., 1996; Atkinson et al., 2004). Fewer studies focused directly on identifying the type of action performed (Dittrich, 1993), however, studies in which point light displays representing different types of action were compared and showed that actions differ with regards to the information they reveal about the actor’s identity (Loula et al., 2005; Sevdalis and Keller, 2009). Recent literature suggests that body motion (e.g., gait) in general contributes significantly to person recognition in real-world scenarios, in particular from a distance or in uncertain viewing conditions, whereas from close-up, the face is the primary cue for recognition (Rice et al., 2013; Hahn et al., 2015). Yovel and O’Toole (2016) provide a framework explaining person recognition in the real world, suggesting that dynamic information, in the form of dynamic identity signatures, plays the central role in binding together information from face, body, and voice into a multi-modal dynamic representation of a person, and that this binding function is the main contribution of the superior temporal sulcus to social cognition. Taken together, these studies corroborate that body motion plays a crucial role in person recognition and that the type of action as well as the action context interacts with this process.

Comparing different types of actions represented as point-light displays, Sevdalis and Keller (2009) found that free dancing resulted in better self-recognition from point-light displays compared to walking and clapping, and suggested that this finding was based on the more pronounced “kinematic fingerprint” of the improvised dance movement compared to other actions. Dance movements often do not involve interactions with objects or persons and have no obvious external goal that can be referred to with respect to its anticipated outcome (e.g., Prinz, 1997; Hommel et al., 2001). Instead, dance movements typically possess internal goals that are related to the movement itself, its trajectory, dynamics, and expression. Schachner and Carey (2013) refer to actions that do not have obvious external goals as “dance-like,” even if these actions are not performed in a dance context. The authors showed that observers tended to interpret actions as intentionally movement-related (and thereby “dance-like”) if they were not able to infer external goals from observing the actions, or if the actions seemed inefficient or inappropriate with respect to any potential external goal. In dance training, movement learning is most commonly practiced by observation of a human model, and observational learning has proved to be the most successful learning mode (Schmidt, 1975, 2003; Blandin and Proteau, 2000; Hodges et al., 2007). Performing movement with closed eyes, however, is considered a meaningful practice in modern and contemporary dance training, as it provides an unusual experience with enhanced perception of kinesthetic, proprioceptive, haptic, and acoustic information. In this study, our aim was to apply the movement-based approach to action and actor recognition provided by the use of point-light displays to dance-like actions that had been acquired in the absence of visual feedback.

Casile and Giese (2006) showed that motor experience of an action (walking) can facilitate the visual recognition of that action, even in the absence of observational learning or visual experience. In their study, they applied a learning paradigm based on verbal and haptic feedback to dissociate visual and motor learning of unusual gait patterns. The results showed that visual recognition of the non-visually learnt material was improved compared to similar but untrained movement material and that recognition performance correlated with the ability to perform the learnt movement. The authors concluded that non-visual motor learning has a direct influence on visual action recognition.

Our aim was to extend the findings by Casile and Giese (2006) to non-cyclic full-body movements other than gait patterns. Additionally, in order to make sure that movement representations were based solely on proprioceptive and kinaesthetic feedback, we used motor actions that were not learnt through haptic guidance or verbal instruction, but created by the participants themselves in the absence of vision. In our study, we investigated to what extent participants were able to recognize movements they had created and performed while they were blindfolded from visual observation of point-light displays. Visual recognition performance of these blindfolded self-created movements was compared to that of learnt (via observation and movement practice) and only observed (without physically moving along) movements, and to unfamiliar movements as control. We expected that the “blind-created” movements could be recognized successfully from visual observation equally well as movements that had been learnt from observation and imitation, based on the multimodal nature of the action representation built up during the creation and execution of the movement. Furthermore, based on previous studies (e.g., Loula et al., 2005; Sevdalis and Keller, 2009), we expected that participants would be able to distinguish between themselves and others as performers of the action equally well for actions they had learnt from observation and actions they had created while being blindfolded. The ability to differentiate between oneself and others on the basis of visual and acoustic action information has been investigated by many authors [see Knoblich and Flach (2003) for review], and rich evidence exists that dynamic visual cues (such as those provided by point-light displays) are particularly well suited for self-recognition (e.g., Troje et al., 2005). Self-other discrimination from sound information representing complex motor actions has also been studied in the absence of visual information for sports (e.g., Murgia et al., 2012; Kennel et al., 2014) and musical contexts (see Sevdalis and Keller, 2014). These findings support the view that action representations stored in memory are based on motor execution and practice, and thereby include individualized information deriving from the performer‘s motor system that can be accessed through different modalities (Flach et al., 2004; Repp and Knoblich, 2004; Repp and Keller, 2010).

## Materials and Methods

### Participants

Nineteen sports students (22–26 years of age, mean 23.4 years; all right handed, four males) participated in this study. Seven out of the 19 students practiced dance or gymnastics regularly, 9 trained ball games (mostly soccer and volleyball), others most practiced sports included tennis, running, and fitness training. All students took part in the same seminar, participation in the experiment was recommended for their own experience but was not necessary for course credit. The students were not informed about the purpose of the movement recording session before taking part in the following point-light experiment. This study was carried out in accordance with the recommendations of the ethics committee of Bielefeld University. A prospective ethics approval was not required in agreement with the institutional institution’s guidelines and national regulations. All subjects gave written informed consent in accordance with the Declaration of Helsinki.

### Procedure

The procedure consisted of two sessions, a movement recording session and a recognition session. During the first session, movement phrases were recorded with groups of students in the biomechanics lab using Vicon motion capture to produce point-light displays as stimuli for the following action recognition experiment. In the second session, each student participated individually in the action recognition experiment. The first session took place on the same day for all participants; the second session was conducted 14–21 days after the first session.

### Action Recording Session

For the first session, students were randomly assigned to six groups of three (in one case four). Two groups entered the biomechanics lab together for a recording session of approximately 1 h, resulting in three recording sessions for all 19 participants. The recording session with two groups took place in the following way: After entering the biomechanics lab, one group was defined by the experimenter as “observers” and instructed to sit on the side of the lab watching the other group attentively and quietly, without moving themselves. The other group was defined as “active group,” and each member of this group were equipped with 15 retro-reflective markers positioned the body (one on each foot, knee, hip, hand, elbow, shoulder; two on the forehead; and one on the sternum). Subsequently, one member of the active group was blindfolded with a sleeping mask, and was lead to the middle of the recording space (approximately 2 × 2 m). The two other members of the active group were standing outside the recording space, with sufficient space around them to move freely and to watch their blindfolded group member. The blindfolded participant was then instructed to start moving and create a short movement phrase that s/he considered novel and unusual, and to repeat it until s/he felt confident (creating movement blindfolded had been tried out in the seminar once before, so this practice was not entirely new to the students, but they had not been informed that this would be done during the movement recording session). The two other students were instructed to watch the “movement designer” and to learn the movement by imitating or marking. After the two partners indicated that they felt confident performing the movement, each member of the group was recorded performing the movement individually three to five times using the Vicon motion capture system. The “movement designer” remained blindfolded throughout until the recording of “his/her” movement with all partners was finished, whereas the “learners” were performing with their eyes open. After the recording of this particular movement, the blindfold was removed from the “movement designer’s” eyes and one of the partners (“learners”) was assigned the new “movement designer.” The procedure was repeated for each member of the “active” group so that everyone took the role of the blindfolded movement designer once, and each member of the active group was recorded performing his/her own and every other member’s movement. Subsequently, the active group and the group of observers swapped roles, the observers were seated on the side of the lab, and the whole procedure was repeated with the new active group.

### Recognition Task

The Vicon recordings were transformed into 2D video clips, with all movements being shown from the same distance and perspective (designated front view). Short clips each containing one full performance of each movement were cut from the footage to produce the stimulus material for the movement recognition task. For stimulus presentation, Presentation^®^ software (14.8) was used. During the experiment, 36 movements were shown once in randomized order. Each movement clip was preceded by a screen with the text “Movement no. x” (with x being the number of the presented clip, counting from 1 to 36) for 2000 ms, a black screen (500 ms) and a fixation cross (500 ms), and followed by a black response screen during which the stimulus presentation was paused. To continue the experiment (with presentation of the next stimulus), the participant had to press the space bar. Each participant was presented 36 video clips showing 12 different movements, each performed by three different persons (one “movement designer” and two “learners”). Six of the movements (18 clips) were familiar (i.e., recorded during the session the participant had taken part in) and six were unfamiliar (recorded during sessions with other groups). For half of the familiar movements (nine clips), the participant had been observer, whereas the other half had been recorded with him/her being “active” (i.e., the participant had performed these movements him/herself, two sighted as learner and one blindfolded).

Each participant performed the movement recognition task individually in a quiet laboratory. The student was instructed to sit in front of the computer screen and watch the displayed point-light clips, and to mark the responses in a paper questionnaire with a pen. After each movement clip, when the stimulus presentation was paused, the participant had to answer two questions by ticking the appropriate boxes, and subsequently to press the space bar to activate the presentation of the next stimulus. The questions that had to be answered for each point-light clip during the experiment (originally in German) were the following:

Question 1: I have ..

- neither watched nor performed this movement;
- only watched this movement, as observer;
- watched, learnt, and performed this movement;
- created and performed, but not watched this movement.

Question 2: The person in the video clip ..

- was me;
- was not me;

### Statistical Analysis

The results are presented with descriptive table and comparisons between rates of answers between categories unadjusted for the dependence of answers made by the same participants are obtained with chi-squared tests. Analyses accounting for multiple responses from the same participants were done using multilevel logistic regression. All analyses were performed using Stata (StataCorp., 2015, Stata Statistical Software: Release 14, College Station, TX, United States: StataCorp LP.).

## Results

### Action Recognition

Answers to Question 1 (action recognition) were categorized into correct answers (i.e., clips correctly assigned to one of the four categories: unfamiliar, observed, learnt, or self-created) and incorrect answers (clips incorrectly assigned). Numbers of answers given for Q1 are displayed in **Table 1**. The distribution of true and false positives per action category answered and of correct answers per action category are provided unadjusted (**Tables 2, 3**) as well as adjusted for multiple responses (**Table 4**).

**Table 1:** Numbers of participants’ answers given for each action category.

Action category Answer given	Unknown	Observed	Learnt	Created	Sum
“Unknown”	318	24	5	5	352
“Observed”	21	141	11	2	175
“Learnt”	3	6	95	1	105
“Created”	0	0	3	49	52
Sum	342	171	114	57	684

**Table 2:** True and false positives per action category answered.

Answer category	Unknown	Observed	Learnt	Created
True positives (*N*, % of answers)	318 (90%)	141 (81%)	95 (90%)	49 (94%)
False positives (*N*, % of answers)	34 (10%)	34 (19%)	10 (10%)	3 (6%)
Total answer	352	175	105	52

**Table 3:** Correct answer per action category.

True action category	Created	Unknown	Observed	Learnt
Correct answers (*N*, % of total)	49 (85%)	318 (92%)	141 (82%)	95 (83%)
Total number of actions	57	342	171	114

**Table 4:** Results of the multilevel logistic regression.

	Odds ratio	95% CI	*p*-value
Model 1	True/false positives		
Answer category			
Created (reference)			
Unknown	0.58	[0.17, 1.98]	0.38
Observed	0.23	[0.07, 0.81]	0.02
Learnt	0.55	[0.14, 2.15]	0.39
Model 2	Correct/false answer		
Action category			
Created (reference)			
Unknown	2.21	[0.92, 5.51]	0.07
Observed	0.75	[0.31, 1.80]	0.53
Learnt	0.80	[0.32, 2.01]	0.65

The unadjusted comparison of the distribution of true and false positive per answer categories (**Table 2**) showed a significant difference. The adjusted comparison (multilevel logistic regression) showed that the odds of a true positive when answering “observed” is 77% lower [OR: 0.23; 95% CI: (0.07, 0.81); *p* = 0.02] than the odds of a true positive when answering “created.” The odds of true positives for the answer categories “unknown” and “learnt” did not significantly differ from the answer category “created” (**Table 4**). With respect to our hypothesis, that means that participants indeed recognized and categorized the actions they had created while blindfolded equally well as actions they had learnt from observation and unfamiliar actions they had neither watched nor performed, whereas they were less successful in recognizing actions they had only observed but not performed themselves.

The distribution of correct answers per scenario categories depends significantly on the category (*p*-value for chi-squared test: 0.01 for **Table 3**). However, the adjusted comparison (multilevel logistic regression) showed no significant difference in the odds giving a correct answer for any type of action category compared to the “created” action category situation (see **Table 4**, Model 2).

### Actor Recognition

Answers to Question 2 (actor recognition) were categorized into correct answers (i.e., clips correctly identified as showing oneself or not showing oneself) and incorrect answers (clips incorrectly identified). Numbers of answers given for Q2 are displayed in **Table 5**.

**Table 5:** Numbers of participants’ answers given for identification of the actor as self or non-self.

Actor category Answer given	Self	Non-self	Sum
“Self”	22 (38.6%)	20 (3.2%)	42
“Non-self”	34 (59.7%)	600 (95.7%)	634
No answer	1 (1.8%)	7 (1.1%)	8
Sum	57 (100%)	627 (100%)	684

Identification of the actor as oneself or not oneself in this task was only meaningful for actions that the participant had performed him- or herself. The next important step therefore is to analyze the results for Question 2 with regards to those for Question 1. This is especially important as participants’ answers to the two questions were not independent of each other, but were given successively for each clip, both in the same trial. When relating self-recognition to action recognition, a difference has to be made between self-recognition with respect to actions the participant had (correctly or incorrectly) assigned to the categories “learnt” or “created” (**Table 6**), and self-recognition with respect to actions that indeed belonged to these categories (**Table 7**). Therefore, in the following, we will differentiate between these two scenarios.

**Table 6:** Numbers of participants’ answers given for identification of the actor as self or non-self for action categories categorized as “created” or “learnt.”

Action category Answer given	Created	Learnt	Sum
“Self”	23 (44%)	14 (13%)	37 (24%)
“Non-self”	29 (56%)	88 (84%)	117 (74%)
No answer	0	3 (3%)	3 (2%)
Sum	52 (100%)	105 (100%)	157 (100%)

**Table 7:** Numbers of participants’ answers given for identification of the actor as self or non-self for action categories answered “created” or “learnt” separately for self true and not true.

	Self is true		Not self is true	
Action category Answer given	Created	Learnt	Created	Learnt
“Self”	14 (70%)	7 (23%)	9 (28%)	7 (9%)
“Non-self”	6 (30%)	22 (73%)	23 (72%)	66 (88%)
No answer	0	1 (3%)	0	2 (3%)
Sum	20 (100%)	30 (100%)	32 (100%)	75 (100%)

Given that the answer to the first question was “learnt” or “created,” the odds of self-recognition was 75% [OR: 0.24, 95% CI (0.11,0.52)] lower for those answering “learnt” compared to those answering “created.” Reducing the comparison to the questions for which “self” was true (50 answers for 19 participants) the odds of self-recognition was 98% [OR: 0.02, 95% CI (0.00, 0.56)] lower for those answering “learnt” compared to those answering “created.” Reducing the comparison to the questions for which “self” was not true (107 answers for 19 participants) the odds of self-recognition was 65% [OR: 0.34, 95% CI (0.12, 0.98)] lower for those answering “learnt” compared to those answering “created.” This means that participants were more likely to recognize themselves as actors (correctly or not) in clips they had assigned to the category “created” than those they had assigned to the category “learnt.” This effect was much stronger in the situation were self was actually true.

## Discussion

In a study with participants who had only basic dance experience, we were interested in the participants’ ability to recognize movement phrases they had experienced through learning from observation and practice, from pure observation, or from improvisation without vision while being blindfolded. We presented our participants with 36 video-clips showing point-light displays of dance-like actions that had been recorded with the same participants during a previous session. The clips showed dance-like movements of four categories: unfamiliar, observed (but not performed), learnt (observed and performed), and self-created while blindfolded (performed, but not observed). Based on previous studies (e.g., Casile and Giese, 2006), we expected that participants would be able to assign the presented movement phrases to the correct categories, independent of the modality of their specific previous experience of that action (visual, kinaesthetic, both, or none); in particular, we expected the recognition accuracy for the blindfolded self-created movement phrases to be on the same level as for the other categories. Results showed that participants assigned movements of all four categories correctly to the respective categories, showing that all three ways of experiencing the movement (observed, learnt, created blindfolded) resulted in an encoding of the movement in long-term memory that was sufficient for recognition (Schmidt, 1975, 2003). Observed movements showed the lowest level of recognition accuracy, whereas the accuracy of assigning blindfolded self-created movements was on the same level as for unfamiliar and learnt movements.

As main finding of this study, the recognition of point-light displays from movements that the participants had created and performed, but never visually experienced, was equally high as for the movements they had learnt through observation and practice, and higher than for the movements they had only observed. This finding corroborates results of a previous study in which participants learnt gait patterns without visual feedback, based only on haptic and verbal cues (Casile and Giese, 2006). The performance of the participants in assigning the visually displayed movements correctly points toward a perceptual equivalence of movements learnt from observation and those created blind-folded during the recording session, as both could be accessed via visual observation of the point-light display equally well. These findings support the idea of an intermodal mapping of kinesthetic and proprioceptive movement representations to the visual domain (Schütz-Bosbach and Prinz, 2007).

According to recent approaches, motor learning and execution is based on the integration of visual, auditory, verbal, proprioceptive, and kinaesthetic information into a holistic multimodal mental representation of the learnt action in long-term memory (Zacks et al., 2007; Barsalou, 2008). Such representations are supposed to comprise declarative and non-declarative memory content that is integrated and updated with every new access and are therefore thought to underlie the physical execution as well as the mental imagery of a motor action, with their internal structure depending on the quality of performance (Land et al., 2013). Nomikou et al. (2016) argue in favor of rich multimodal representations continuously developed through and for action and interaction, suggesting that such representations are built early during development by acting and interacting in the physical and social world. Such representations have to be dynamic in nature to capture temporal progression and allow for prediction; they need to express temporal relations allowing for synchronization and co-occurrence as prerequisites for social behaviors. Evidence exists, in particular for audio–visual information, that multimodal action representations are transferable between sensory modalities and can even be accessed through senses that were not actively involved in the process of action acquisition (Rosenblum et al., 2017). Rosenblum et al. (2017) propose that the architecture of the brain implies perceptual parity between the senses, and that cross-sensory integration occurs completely and early in the perceptual stream. The authors argue in favor of task rather than sensory modality as primary organizing principle, and suggest that perceptual learning might involve extracting amodal primitives that are not specifically tied to sensory modalities, therefore perceptual learning within the same task context should be transferable between senses. This argument provides explanatory ground with regards to the results of the present study in which participants showed that they were able to exploit movement information gained through physical execution without visual feedback for a visual recognition task. In real-world motor learning tasks in sports and dance, information from other sensory modalities such as action-related sound contributes significantly to motor learning (e.g., Camponogara et al., 2017; Sors et al., 2017). Camponogara et al. (2017) showed that expert basketball players were able to infer opponents’ movement intentions from action-based sound more accurately than novices, by picking up action-specific movement information and using it to anticipate the opponent’s future position. The authors suggest that the experts pick up relevant kinematic features such as velocity, trajectory, and position of deceptive movements through structural and transformational invariants of the movement sounds by directly mapping sound characteristics onto action intentions. These findings are supported by fMRI results showing that sports experts display specific activation in brain areas involved in action planning when passively listening to task-relevant sounds from their own area of expertise, but not in response to irrelevant sounds (Woods et al., 2014).

With regards to the creating movement task applied in the current study, it cannot be ruled out that the participants, while being blindfolded, created mental images of the performed action using visual imagery. As this is not unlikely, it might have been interesting to investigate the influence of cognitive strategies on action recognition, for example by means of a *post hoc* questionnaire or interview. In dance, mental imagery is applied for different purposes including the rehearsal, creation, and interpretation of movement and the preparation or recreation of the body (Hanrahan and Vergeer, 2001; Nordin and Cumming, 2007), and dance training has been found to increase the efficiency of imagery techniques (Golomer et al., 2008; Fink et al., 2009). Even participants without dance training experience might have used visual imagery during the experimental task to compensate for the lack of visual feedback.

The finding that recognition accuracy for observed movements was below that of movements learnt through observation and practice supports the notion that action execution is generally more beneficial for learning than observation alone (e.g., Badets et al., 2006). In sports and dance training, the learning of dance-like actions (see Schachner and Carey, 2013) is most commonly practiced in the form of observational learning from a visual model, typically augmented by verbal comments as teacher feedback (Wulf and Prinz, 2001) and supported by simultaneous movement execution or marking (Kirsh, 2011; Warburton et al., 2013). The often-observed superiority of combined motor and visual learning, compared to visual learning alone, can be explained with reference to the integration of multisensory information during action acquisition (Land et al., 2013), by stating that the participation of more sensory modalities in the learning process might result in a richer representation that involves more complementary information and therefore leads to a better learning outcome. Even though the majority of studies supports the view that physical execution results in better learning than mere observation, evidence against such an enactment effect has also been found, in particular for complex “real-world” type tasks involving longer action sequences (von Stülpnagel et al., 2016a,b). Findings by Allerdissen et al. (2017) suggest that it might not be the mere redundancy of information that enhances learning success in multimodal conditions, but rather the contribution of different modalities providing slightly different information that is then integrated in a meaningful way, and that the ability to integrate relevant information into a consistent action representation and omit irrelevant or contradictory information can be considered a feature of domain-specific expertise. Plenty of evidence exists that auditory information is more accurate than vision with regards to temporal action features and that action control therefore relies more strongly on sound if timing, speed, or rhythm is crucial (e.g., Repp and Penel, 2002), which is of particular relevance in sports (Camponogara et al., 2017; Sors et al., 2017). Studies using audio-based interventions in sports support the view that auditory information is more pertinent than visual information with regards to rhythmic movement features and precisely timed actions (Sors et al., 2015). A study with tap dancers showed that temporal properties of rhythmic dance movement can be better perceived through auditory than visual stimuli (Murgia et al., 2017). In this study, experts’ accuracy in recognizing dance steps was higher than non-dancers’ in the auditory domain, and in the auditory than in the visual domain.

As a second point of interest, we investigated self-recognition from the point-light displays presented in the recognition task by asking the participants to identify the actor as self or non-self. Previous studies had shown that people can easily distinguish between themselves, familiar persons, and strangers from point-light displays of different types of actions (e.g., Loula et al., 2005).

Discrimination between one’s own compared to another’s motor actions on the basis of action-based auditory information has been proved successful for different sports (Murgia et al., 2012; Kennel et al., 2014), and EEG evidence has supported these findings by reporting activation of an evaluation network for agent identification through action-related sound stimuli (Justen et al., 2014). Even though the rhythmic structure has been identified as relevant factor, self recognition from action-based sound does not depend on rhythmic features exclusively, but on a more complex auditory “gestalt” (Kennel et al., 2014). Sevdalis and Keller (2009) found that free dancing resulted in better self-recognition from point-light displays compared to walking and clapping, and argued in favor of a more pronounced “kinematic fingerprint” of the improvised full-body motor action. Loula et al. (2005) also observed that participants identified themselves and familiar persons successfully from point-light displays of dancing, boxing, and playing ping-pong, but failed to reach chance level for displays of walking and running. Mitchell and Curry (2016), in contrast, found that participants identified themselves above chance level from walking point-light walkers presented from different perspectives. In our study, participants did not identify themselves correctly above chance level if only the video clips of self-created and learnt movements are taken into account (self-identification in clips showing unfamiliar or only observed movements would not make much sense in the given context). Only 22 out of 57 clips (38.6%) showing the participant him- or herself performing a self-created of learnt movement were identified as “self,” and 20 clips were erroneously identified as “self.”

Furthermore, we found an interesting interaction between action and actor recognition: participants were more likely to identify the actor as “self” in clips they had assigned to the category “created” than in clips they had assigned to the category “learnt.” This effect was much stronger in the situation if the “self” judgment was actually true. These results show that self-recognition and action recognition influenced each other and that categorization of a movement as “learnt” or “self-created” had a biasing effect on actor identification, which points toward a significant role for agency for self-recognition (see Knoblich and Flach, 2003; Jeannerod and Pacherie, 2004).

Previous studies had shown that actor recognition and action recognition are not independent from each other in different conditions, for example that knowing an actor’s identity and intention can influence action perception (e.g., Knoblich and Sebanz, 2006; Sebanz et al., 2006). Ferstl et al. (2017) suggest that neural mechanisms might exist that link actor information to action information by encoding actor identity on the basis of specific cues (facial features, clothing, posture) in service of action prediction. They claim that action recognition should be sensitive to actor identity for reasons of ecological validity, as information about the actor is fundamental for understanding observed actions. Schütz-Bosbach et al. (2006) showed that observing others’ actions facilitated the motor system, whereas observing one’s own actions rather suppressed motor activation. Based on their results, the authors argued strongly against agent-neutral action representations, suggesting that neural mechanisms underlying action observation are intrinsically social. These studies support the view that action recognition is influenced by actor recognition, however, in the present study, it could be claimed that we found a reverse effect, namely that actor recognition is influenced by action recognition. In this regard, the order in which participants were asked to identify action and actor the recognition task might be relevant. For each presented video clip, the participant had to answer two questions within the same trial, before the next clip was shown; in each trial, action recognition (or action categorization) came before actor recognition (or action identification). This way, participants were judging the presented movement first on its own merit, however, it cannot be excluded that the action recognition thereby had a priming effect on actor recognition, which might have caused or enhanced the observed interaction bias. First identifying a presented movement as self-created might have influenced the “self or other” decision by shifting it toward “self,” which is reflected by the results. It would be interesting to know if the same interaction had been found if the questions had been asked in the opposite order (self-identification before action categorization), or if the two questions had been asked separately in different blocks.

Another limitation of the presented experiment might be seen in the choice of participants. This study was conducted with 19 sports students whose dance experience differed (seven practiced dance or gymnastics regularly, whereas the others practiced other types of sports). Even though none of the participants reached professional level in dance, their different experience might have affected their individual approach to learning and creating movement (however, none of the movement phrases created in the first session was particularly complex or too difficult to be picked up easily by dance novices). Repeating the experiment with more homogeneous expertise-based groups (professional dancers vs. non-dancers) could provide relevant novel insights regarding these aspects.

## Conclusion

The results of the presented study support findings of a direct influence of motor experience on visual action perception and recognition for actions that have been learnt without visual feedback. They extend previous results (Casile and Giese, 2006), to dance-like actions that have been acquired exclusively through movement exploration and practice, in the absence of vision and without haptic or verbal feedback. The “blind” execution and creation of full-body actions (as it is typically applied in contemporary dance training) obviously results in a multimodal representation that can be accessed via visual cues, despite the lack of visual experience. Furthermore, the results corroborate that agency plays a significant role for self-identification, which adds new aspects to perspectives taken in social cognition contexts (Ferstl et al., 2017).

## Author Contributions

BB planned and conducted the study and pre-analyzed the data. OS analyzed and interpreted the data. Both authors contributed equally to the manuscript.

## Conflict of Interest Statement

The authors declare that the research was conducted in the absence of any commercial or financial relationships that could be construed as a potential conflict of interest.

## References

[B1] Allerdissen M. Güldenpenning I. Schack T. Bläsing B. (2017). Recognizing fencing attacks from auditory and visual information: a comparison between expert fencers and novices. *Psychol. Sport Exerc.* 31 123–130. 10.1016/j.psychsport.2017.04.009

[B2] Atkinson A. P. Dittrich W. H. Gemmell A. J. Young A. W. (2004). Emotion perception from dynamic and static body expressions in point-light and full-light displays. *Perception* 33 717–746. 10.1068/p5096 15330366

[B3] Badets A. Blandin Y. Shea C. H. (2006). Intention in motor learning through observation. *Q. J. Exp. Psychol.* 59 377–386. 10.1080/02724980443000773 16618640

[B4] Barsalou L. W. (2008). Grounded cognition. *Annu. Rev. Psychol.* 59 617–645. 10.1146/annurev.psych.59.103006.093639 17705682

[B5] Blake R. Shiffrar M. (2007). Perception of human motion. *Annu. Rev. Psychol.* 58 47–73. 10.1146/annurev.psych.57.102904.190152 16903802

[B6] Blandin Y. Proteau L. (2000). On the cognitive basis of observational learning: development of mechanisms for the detection and correction of errors. *Q. J. Exp. Psychol. A* 53 846–867. 10.1080/713755917 10994232

[B7] Camponogara I. Rodger M. Craig C. Cesari P. (2017). Expert players accurately detect an opponent’s movement intentions through sound alone. *J. Exp. Psychol. Hum. Percept. Perform.* 43 348–359. 10.1037/xhp0000316 27831718

[B8] Casile A. Giese M. A. (2006). Nonvisual motor training influences biological motion perception. *Curr. Biol.* 16 69–74. 10.1016/j.cub.2005.10.071 16401424

[B9] Cutting J. E. Kozlowski L. T. (1977). Recognizing friends by their walk: gait perception without familiarity cues. *Bull. Psychon. Soc.* 9 353–356. 10.3758/BF03337021

[B10] Dittrich W. H. (1993). Action categories and the perception of biological motion. *Perception* 22 15–22. 10.1068/p220015 8474831

[B11] Dittrich W. H. Troscianko T. Lea S. E. Morgan D. (1996). Perception of emotion from dynamic point-light displays represented in dance. *Perception* 25 727–738. 10.1068/p250727 8888304

[B12] Ferstl Y. Bülthoff H. de la Rosa S. (2017). Action recognition is sensitive to the identity of the actor. *Cognition* 166 201–206. 10.1016/j.cognition.2017.05.036 28582683

[B13] Fink A. Graif B. Neubauer A. C. (2009). Brain correlates underlying creative thinking: EEG alpha activity in professional vs. novice dancers. *Neuroimage* 46 854–862. 10.1016/j.neuroimage.2009.02.036 19269335

[B14] Flach R. Knoblich G. Prinz W. (2004). Recognizing one’s own clapping: the role of temporal cues. *Psychol. Res.* 69 147–156. 10.1007/s00426-003-0165-2 15095091

[B15] Golomer E. Bouillette A. Mertz C. Keller J. (2008). Effects of mental imagery styles on shoulder and hip rotations during preparation of pirouettes. *J. Mot. Behav.* 40 281–290. 10.3200/JMBR.40.4.281-290 18628105

[B16] Hahn C. A. O’Toole A. J. Phillips P. J. (2015). Dissecting the time course of person recognition in natural viewing environments. *Br. J. Psychol.* 107 117–134. 10.1111/bjop.12125 25752865

[B17] Hanrahan C. Vergeer I. (2001). Multiple uses of mental imagery by professional modern dancers. *Imagin. Cogn. Pers.* 20 231–255. 10.2190/RLBE-XQK9-C65F-X05B

[B18] Hodges N. J. Williams A. M. Hayes S. J. Breslin G. (2007). What is modelled during observational learning? *J. Sport Sci.* 25 531–545. 10.1080/02640410600946860 17365540

[B19] Hommel B. Muesseler J. Aschersleben G. Prinz W. (2001). The theory of event coding (TEC): a framework for perception and action planning. *Behav. Brain Sci.* 24 849–878. 10.1017/S0140525X01000103 12239891

[B20] Jeannerod M. Pacherie E. (2004). Agency, simulation and self-identification. *Mind Lang.* 19 113–146. 10.1111/j.1468-0017.2004.00251.x

[B21] Johansson G. (1973). Visual perception of biological motion and a model for its analysis. *Percept. Psychophys.* 14 201–211. 10.3758/BF03212378

[B22] Justen C. Herbert C. Werner K. Raab M. (2014). Self vs. other: neural correlates underlying agent identification based on unimodal auditory information as revealed by electrotomography (sLORETA). *Neuroscience* 259 25–34. 10.1016/j.neuroscience.2013.11.042 24295635

[B23] Kennel C. Pizzera A. Hohmann T. Schubotz R. I. Murgia M. Agostini T. (2014). The perception of natural and modulated movement sounds. *Perception* 43 796–804. 10.1068/p7643 25549509

[B24] Kirsh D. (2011). “Creative cognition in choreography,” in *Proceedings of 2nd International Conference on Computational Creativity 2011.* (San Diego, CA: University of California).

[B25] Knoblich G. Flach R. (2003). Action identity: evidence from self-recognition, prediction, and coordination. *Conscious. Cogn.* 12 620–632. 10.1016/S1053-8100(03)00070-9 14656505

[B26] Knoblich G. Sebanz N. (2006). The social nature of perception and action. *Curr. Dir. Psychol. Sci.* 15 99–104. 10.1111/j.0963-7214.2006.00415.x

[B27] Kozlowski L. T. Cutting J. E. (1977). Recognizing the sex of a walker from a dynamic point-light display. *Percept. Psychophys.* 21 575–580. 10.3758/BF03198740

[B28] Land W. M. Volchenkov D. Bläsing B. Schack T. (2013). From action representation to action execution: exploring the links between cognitive and biomechanical levels of motor control. *Front. Comput. Neurosci.* 7:127. 10.3389/fncom.2013.00127 24065915 PMC3776155

[B29] Loula F. Prasad S. Harber K. Shiffrar M. (2005). Recognizing people from their movement. *J. Exp. Psychol. Hum. Percept. Perform.* 31 210–220. 10.1037/0096-1523.31.1.210 15709874

[B30] Mitchell R. W. Curry C. (2016). Self-recognition and other-recognition in point-light displays. *Open J. Philos.* 6 42–50. 10.4236/ojpp.2016.61005

[B31] Murgia M. Hohmann T. Galmonte A. Raab M. Agostini T. (2012). Recognising one’s own motor actions through sound: the role of temporal factors. *Perception* 41 976–987. 10.1068/p7227 23362674

[B32] Murgia M. Prpic V. Jenny O. McCullagh P. Santoro I. Galmonte A. (2017). Modality and perceptual-motor experience influence the detection of temporal deviations in tap dance sequences. *Front. Psychol.* 8:1340. 10.3389/fpsyg.2017.01340 28824516 PMC5539223

[B33] Nomikou I. Schilling M. Heller V. Rohlfing K. J. (2016). Language-at all times. *Interact. Stud.* 17 120–145. 10.1075/is.17.1.06nom

[B34] Nordin S. M. Cumming J. (2007). Where, when, and how: a quantitative account of dance imagery. *Res. Q. Exerc. Sport* 78 390–395. 10.1080/02701367.2007.10599437 17941544

[B35] Pollick F. E. Kay J. W. Heim K. Stringer R. (2005). Gender recognition from point-light walkers. *J. Exp. Psychol. Hum. Percept. Perform.* 31 1247–1265. 10.1037/0096-1523.31.6.1247 16366787

[B36] Prinz W. (1997). Perception and action planning. *Eur. J. Cogn. Psychol.* 9 129–154. 10.1080/713752551

[B37] Rice A. Phillips P. J. O’Toole A. (2013). The role of the face and body in unfamiliar person identification. *Appl. Cogn. Psychol.* 27 761–768. 10.1002/acp.2969

[B38] Repp B. H. Keller P. E. (2010). Self versus other in piano performance: detectability of timing perturbations depends on personal playing style. *Exp. Brain Res.* 202 101–110. 10.1007/s00221-009-2115-8 20012533

[B39] Repp B. H. Knoblich G. (2004). Perceiving action identity: how pianists recognize their own performances. *Psychol. Sci.* 15 604–609. 10.1111/j.0956-7976.2004.00727.x 15327631

[B40] Repp B. H. Penel A. (2002). Auditory dominance in temporal processing: new evidence from synchronization with simultaneous visual and auditory sequences. *J. Exp. Psychol. Hum. Percept. Perform.* 28 1085–1099. 10.1037/0096-1523.28.5.1085 12421057

[B41] Rosenblum L. D. Dias J. W. Dorsi J. (2017). The supramodal brain: implications for auditory perception. *J. Cogn. Psychol.* 29 65–87. 10.1111/ejn.12140 23383863

[B42] Schachner A. Carey S. (2013). Reasoning about ‘irrational’actions: when intentional movements cannot be explained, the movements themselves are seen as the goal. *Cognition* 129 309–327. 10.1016/j.cognition.2013.07.006 23958932

[B43] Schmidt R. A. (1975). A schema theory of discrete motor skill learning. *Psychol. Rev.* 82 225–260. 10.1037/h0076770

[B44] Schmidt R. A. (2003). Motor schema theory after 27 years: reflections and implications for a new theory. *Res. Q. Exerc. Sport* 74 366–375. 10.1080/02701367.2003.10609106 14768837

[B45] Schütz-Bosbach S. Mancini B. Aglioti S. M. Haggard P. (2006). Self and other in the human motor system. *Curr. Biol.* 16 1830–1834. 10.1016/j.cub.2006.07.048 16979561

[B46] Schütz-Bosbach S. Prinz W. (2007). Perceptual resonance: action-induced modulation of perception. *Trends Cogn. Sci.* 11 349–355. 10.1016/j.tics.2007.06.005 17629544

[B47] Sebanz N. Bekkering H. Knoblich G. (2006). Joint action: bodies and minds moving together. *Trends Cogn. Sci.* 10 70–76. 10.1016/j.tics.2005.12.009 16406326

[B48] Sevdalis V. Keller P. E. (2009). Self-recognition in the perception of actions performed in synchrony with music. *Ann. N. Y. Acad. Sci.* 1169 499–502. 10.1111/j.1749-6632.2009.04773.x 19673830

[B49] Sevdalis V. Keller P. E. (2014). Know thy sound: perceiving self and others in musical contexts. *Acta Psychol.* 152 67–74. 10.1016/j.actpsy.2014.07.002 25113128

[B50] Sors F. Murgia M. Santoro I. Agostini T. (2015). Audio-based interventions in sport. *Open Psychol. J.* 8 212–219. 10.2174/1874350101508010212

[B51] Sors F. Murgia M. Santoro I. Prpic V. Galmonte A. Agostini T. (2017). The contribution of early auditory and visual information to the discrimination of shot power in ball sports. *Psychol. Sport Exerc.* 31 44–51. 10.1016/j.psychsport.2017.04.005

[B52] Thornton I. M. (2006). “Biological motion: point-light walkers and beyond,” in *Human Body Perception from the Inside Out*, eds Knoblich G. Thornton M. Grosjean M. Shiffrar M. (New York, NY: Oxford University Press),271–305.

[B53] Troje N. F. Westhoff C. Lavrov M. (2005). Person identification from biological motion: effects of structural and kinematic cues. *Percept. Psychophys.* 67 667–675. 10.3758/BF03193523 16134460

[B54] von Stülpnagel R. Schult J. C. Richter C. Steffens M. C. (2016a). Cognitive costs of encoding novel natural activities: can “learning by doing” be distracting and deceptive? *Q. J. Exp. Psychol.* 69 1545–1563. 10.1080/17470218.2015.1087581 26325343

[B55] von Stülpnagel R. Steffens M. C. Schult J. C. (2016b). Memory for five novel naturalistic activities: no memory recall advantage for enactment over observation or pictorial learning. *J. Articles Support Null Hypothesis* 12 1–16.

[B56] Warburton E. C. Wilson M. Lynch M. Cuykendall S. (2013). The cognitive benefits of movement reduction: evidence from dance marking. *Psychol. Sci.* 24 1732–1739. 10.1177/0956797613478824 23863756

[B57] Woods E. A. Hernandez A. E. Wagner V. E. Beilock S. L. (2014). Expert athletes activate somatosensory and motor planning regions of the brain when passively listening to familiar sports sounds. *Brain Cogn.* 87 122–133. 10.1016/j.bandc.2014.03.007 24732956

[B58] Wulf G. Prinz W. (2001). Directing attention to movement effects enhances learning: a review. *Psychon. Bull. Rev.* 8 648–660. 10.3758/BF03196201 11848583

[B59] Yovel G. O’Toole A. J. (2016). Recognizing people in motion. *Trends Cogn. Sci.* 20 383–395. 10.1016/j.tics.2016.02.005 27016844

[B60] Zacks J. M. Speer N. K. Swallow K. M. Braver T. S. Reynolds J. R. (2007). Event perception: a mind-brain perspective. *Psychol. Bull.* 133 273–293. 10.1037/0033-2909.133.2.273 17338600 PMC2852534

